# Adults with perinatally acquired HIV in low‐ and middle‐income settings: time for a generational shift in HIV care and global guidance

**DOI:** 10.1002/jia2.26338

**Published:** 2024-07-22

**Authors:** Annette H. Sohn, Mary‐Ann Davies

**Affiliations:** ^1^ TREAT Asia amfAR – The Foundation for AIDS Research Bangkok Thailand; ^2^ Centre for Infectious Disease Epidemiology and Research School of Public Health University of Cape Town Cape Town South Africa

1

Paediatricians caring for children living with HIV started sounding alarm bells about their poorer clinical outcomes from the very beginnings of the HIV epidemic. They were routinely diagnosed late and with advanced disease, lacked appropriate antiretroviral formulations for treatment and their viruses became resistant to these regimens more rapidly, and suffered higher mortality rates [1]. As those who survived became adolescents, they experienced long‐term side effects of their treatment, increased risks for non‐communicable diseases, and the social and mental health impacts of stigma, discrimination and orphanhood [2, 3]. Adults with perinatal HIV are now being managed with limited standards around optimal care delivery.

UNAIDS estimates that there were about 660,000 (560,000–760,000) adults 20–24 years of age living with perinatally acquired HIV in 2023, 88% of whom were in Africa (UNAIDS 2024 epidemiologic estimates). In Asia, Thailand was one of the earliest countries to begin a national HIV treatment programme for children in the mid‐2000s, and now ∼1800 adults >18 years of age are estimated to be living with perinatal HIV—with the oldest in their third decade (Thai National AIDS Program, 2022 data). Although many national surveillance systems do not capture the mode of HIV acquisition, data on age at diagnosis are sufficient to identify those with early exposure to HIV and antiretroviral therapy and track them into adulthood. There is an urgent need for evidence‐based guidelines for the treatment and care of adults with perinatal HIV that can be implemented in low‐ and middle‐income country (LMIC) settings, as well as standardized provider training to effectively implement them.

In high‐income contexts like the United States (US) and the United Kingdom (UK), most of those with perinatal HIV have already transitioned into adult life and HIV care, with some entering their fifth decade [4, 5]. Data on their outcomes are sobering. In the US, by age 30, the cumulative incidence of type‐2 diabetes among those with perinatally acquired HIV was 19%, 22% for hypertension and 25% for chronic kidney disease [6]. A modelling study estimated that life expectancy in US male youth with perinatal HIV was 10.4 years lower and in female youth 11.8 years lower than their HIV‐negative peers [7]. A UK study showed that a lower nadir CD4 count in early childhood had an ongoing negative impact on CD4 by age 20 [8].

Research from LMICs has reflected increased risks for adolescents with perinatal HIV that similarly bode poorly for their health outcomes as adults. Cohorts from South Africa and Thailand have reported bone, cardiac, neurocognitive or respiratory impairments [2, 9]. The lack of prior access to human papillomavirus vaccines has put the current generation of young adults at risk for anogenital cancers (e.g. cervical) [10]. Early reports have raised concerns around reproductive health outcomes and the risk of vertical transmission among those with perinatal HIV, due in part to the greater challenges in managing antiretroviral therapy during pregnancy in those with a history of prior treatment failure and drug resistance [11].

Unique to all children with chronic diseases who survive to adulthood is the need to transition from paediatric to adult providers. Although many younger and older adults with perinatal HIV have sought ways to remain in “paediatric HIV” care into their mid‐20s, there is a point at which this becomes suboptimal for their care in terms of expertise around adult‐onset diseases and sexual and reproductive health [12]. Transition research has largely focused on the clinical outcomes and perspectives of transitioning youth, but there is less documentation of the preparedness of adult HIV providers to take on their management.

Instead, we have multiple reports on their largely poorer outcomes in terms of viral load suppression, retention in care and mortality after transition [13, 14]. While provider preparedness is not a risk factor for suboptimal clinical outcomes that has been explicitly measured, it is hard to imagine that this is not a factor. Even where primary HIV providers provide care to patients of all ages, there are shifts in how they are managed and expectations around responsibility for care when age‐ and maturity‐related transitions occur.

In addition to studying HIV provider perspectives in LMICs, there is a need for provider training to help them better meet the needs of younger and older adults with perinatal HIV. Notably, models for this type of care are available for those with developmental disabilities and congenital heart disease. The latter even has certification processes for cardiologists seeking to provide “adult congenital” care, together with clinical practice guidelines (American Heart Association and American College of Cardiology, European Society of Cardiology) [15]. There is no formalized process to train and support HIV providers who become responsible for these adults who have survived childhood and adolescence with HIV.

To facilitate provider preparedness, guidelines are needed for managing screening and treatment interventions that can be applied in LMICs. Global HIV treatment guidelines have been an essential part of the HIV response. National programmes have confidence in the rigorous approaches taken to formulate this guidance, which has primarily been through the World Health Organization, and routinely adopted them. With the increasing numbers of adolescents and young adults transitioning to adult HIV providers and care clinics each year, comprehensive guidance would help to set standards for the type of care adults with perinatal HIV receive.

The public health approach is geared towards having all people with HIV being managed under the same guidelines. While this is usually appropriate, we need to couple that with differentiated service delivery that centres on the needs of the individual. Those diagnosed early in life with longer HIV exposure at key developmental stages will have different care needs than those who acquired HIV later in life.

The warning signs are clear that care for adults with perinatal HIV will become increasingly complex. They may require earlier screening and clinical interventions for non‐communicable diseases than their age‐matched peers. The impacts of living with a stigmatized disease from birth also will mean providers cannot solely focus on antiretroviral therapy and laboratory tests to address their needs. Global guidelines would be a critical step in establishing standards for provider training and comprehensive care delivery.

## COMPETING INTERESTS

AHS and M‐AD receive funding to their institutions from ViiV Healthcare.

## AUTHORS’ CONTRIBUTIONS

AHS drafted the Viewpoint, M‐AD provided a critical review and both approved the final version.

## References

[1] Desmonde S , Ciaranello AL , Malateste K , Musick B , Patten G , Vu AT , et al. Age‐specific mortality rate ratios in adolescents and youth aged 10–24 years living with perinatally versus nonperinatally acquired HIV. AIDS. 2021;35(4):625–632. 33252479 10.1097/QAD.0000000000002765 PMC7904586

[2] Frigati LJ , Brown K , Mahtab S , Githinji L , Gray D , Zühlke L , et al. Multisystem impairment in South African adolescents with perinatally acquired HIV on antiretroviral therapy (ART). J Int AIDS Soc. 2019;22(8):e25386. 31441211 10.1002/jia2.25386 PMC6706702

[3] Sohn AH , Singtoroj T , Chokephaibulkit K , Lumbiganon P , Hansudewechakul R , Gani YM , et al. Long‐term post‐transition outcomes of adolescents and young adults living with perinatally and non‐perinatally acquired HIV in Southeast Asia. J Adolesc Health. 2023;72(3):471–479. 36535867 10.1016/j.jadohealth.2022.10.021 PMC11772534

[4] Foster C , Ayers S , McDonald S , Frize G , Chhabra S , Pasvol TJ , et al. Clinical outcomes post transition to adult services in young adults with perinatally acquired HIV infection: mortality, retention in care and viral suppression. AIDS. 2020;34(2):261–266. 31651427 10.1097/QAD.0000000000002410

[5] Henderson M , Fidler S , Foster C. Adults with perinatally acquired HIV; emerging clinical outcomes and data gaps. Trop Med Infect Dis. 2024;9(4):74. 38668535 10.3390/tropicalmed9040074 PMC11053933

[6] Haw NJL , Lesko CR , Ng DK , Lam J , Lang R , Kitahata MM , et al. Incidence of non‐AIDS defining comorbidities among young adults with perinatally‐acquired HIV in North America, 2000–2019. AIDS. 2024;38(9):1366–1374. 38507583 10.1097/QAD.0000000000003892 PMC11211058

[7] Neilan AM , Ufio OL , Brenner IR , Flanagan CF , Shebl FM , Hyle EP , et al. Projected life expectancy for adolescents with HIV in the US. JAMA Health Forum. 2024;5(5):e240816. 38728022 10.1001/jamahealthforum.2024.0816 PMC11087843

[8] Castro H , Sabin C , Collins IJ , Okhai H , Schou Sandgaard K , Prime K , et al. Evolution of CD4 T‐cell count with age in a cohort of young people growing up with perinatally acquired human immunodeficiency virus. Clin Infect Dis. 2024;78(3):690–701. 37820036 10.1093/cid/ciad626 PMC10954325

[9] Sudjaritruk T , Bunupuradah T , Aurpibul L , Kosalaraksa P , Kurniati N , Prasitsuebsai W , et al. Adverse bone health and abnormal bone turnover among perinatally HIV‐infected Asian adolescents with virological suppression. HIV Med. 2017;18(4):235–244. 27477214 10.1111/hiv.12418

[10] Phanuphak N , Teeraananchai S , Hansudewechakul R , Gatechompol S , Chokephaibulkit K , Dang HLD , et al. Incidence and persistence of high‐risk anogenital human papillomavirus infection among female youth with and without perinatally acquired human immunodefiency virus infection: a 3‐year observational cohort study. Clin Infect Dis. 2020;71(8):e270–e280. 31768522 10.1093/cid/ciz1143 PMC7755021

[11] Anderson K , Mutemaringa T , Technau KG , Johnson LF , Braithwaite K , Mokotoane E , et al. The next generation: pregnancy in adolescents and women living with perinatally acquired HIV in South Africa. S Afr Med J. 2021;111(3):260–264. 33944749 10.7196/SAMJ.2021.v111i3.14987 PMC8847806

[12] Kim S , Kim SH , McDonald S , Fidler S , Foster C. Transition to adult services—a positive step. AIDS Care. 2017;29(7):885–889. 27973920 10.1080/09540121.2016.1268672

[13] Asad H , Collins IJ , Goodall RL , Crichton S , Hill T , Doerholt K , et al. Mortality and AIDS‐defining events among young people following transition from paediatric to adult HIV care in the UK. HIV Med. 2021;22(8):631–640. 33939876 10.1111/hiv.13096 PMC8612219

[14] Ounchanum P , Aurpibul L , Teeraananchai S , Lumbiganon P , Songtaweesin WN , Sudjaritruk T , et al. High mortality in adolescents and young adults with perinatally‐acquired HIV in Thailand during the transition to adulthood. AIDS Care. 2024:1–10. 10.1080/09540121.2024.2325100 PMC11518584 38447043

[15] Egidy Assenza G , Krieger EV , Baumgartner H , Cupido B , Dimopoulos K , Louis C , et al. AHA/ACC vs ESC guidelines for management of adults with congenital heart disease: JACC guideline comparison. J Am Coll Cardiol. 2021;78(19):1904–1918. 34736567 10.1016/j.jacc.2021.09.010

